# Application of the Products from the Maillard Reaction of Polyglutamic Acid and Glucose to Prepare Colored and Bioactive Silk

**DOI:** 10.3390/polym10060648

**Published:** 2018-06-10

**Authors:** Wen Zhang, Ren-Cheng Tang

**Affiliations:** National Engineering Laboratory for Modern Silk, College of Textile and Clothing Engineering, Soochow University, 199 Renai Road, Suzhou 215123, China; wzhang0219@stu.suda.edu.cn

**Keywords:** silk, antibacterial activity, antioxidant activity, Maillard reaction, polyglutamic acid, glucose, dyeing

## Abstract

In this work, the Maillard reaction of polyglutamic acid (PGA) and glucose (Glc) was studied, and its functional, polymeric, and colored products were used to dye silk fiber with the aim of imparting bioactivities to silk. The UV–Vis spectroscopic analysis, which was employed to monitor the reaction, revealed the rapid formation of yellowish-brown products at pH 12 and 90 °C, and the great impact of glucose content on the quantity of the products. The FT-IR analysis validated the formation of melanoidin colorants. The silk fiber dyed with the PGA/Glc reaction products at pH 3 displayed a yellowish-brown color, and had very good wash and rub fastness, but poor light fastness. The incorporation of the UV-absorbing moiety into the PGA/Glc reaction products enhanced their light stability. The SEM analysis revealed that the dyed silk fiber was covered by polymeric substances. The dyed silk exhibited durable antibacterial activity against *Staphylococcus aureus* and *Escherichia coli*, and good antioxidant activity. This research expands the application field of the Maillard reaction and provides a novel and eco-friendly approach to prepare the colored and bioactive silk materials.

## 1. Introduction

The Maillard reaction is a non-enzymatic browning reaction between amino-containing compounds and reducing sugars. This reaction involves a series of reaction steps including sugar-amine condensation, Amadori rearrangement, sugar dehydration, sugar fragmentation, amino acid degradation, aldehyde-amine condensation, formation of heterocyclic nitrogen compounds, etc., which produce dark brown to black colored polymers and copolymers, known as melanoidins [1]. It is usually considered that melanoidins are predominantly responsible for the characteristic brown color products [2]. Melanoidins formed at the final stage of a Maillard reaction are generally anionic, heterogeneous, and nitrogen-containing condensation products [2,3].

In the food industry, the Maillard reaction, as a nontoxic and biochemical technology, is widely applied for providing the color and flavor to manufactured foods, e.g., coffee and bakery products [1]. In the textile field, the Maillard reaction of glucose, xylose, dextrose, and galactose with amino groups in wool fiber has been applied to enhance the uptake of reactive and acid dyes [4]. In the leather field, carbohydrates are used as retanning agents to increase the affinity of reactive dyes to crust leather; to achieve this purpose, the modified Maillard reaction is employed, where crust leather is firstly treated by carbohydrates and then oxidized by potassium iodate [5]. More recently, the Maillard reaction has been applied to the in situ coloration of wool, silk, and nylon fibers with various reducing sugars, which imparts yellow and orange-brown colors, as well as good antibacterial performance to these fibers [6,7]. However, a serious issue for this method is the very slow coloration reaction speed as it usually requires at least 8 h to produce brown coloration for wool, silk, and nylon fibers [6]. In order to shorten the coloration time of wool fibers, the glycerol oxides obtained from low-cost glycerol by the Fenton reaction have been employed [8].

Polyglutamic acid (PGA) is a non-toxic, biodegradable, and biocompatible polymer, and has a range of attractive applications in the fields of food, agriculture, biomaterials, medicine, and the environment [9]. In the present study, the Maillard reaction of PGA and glucose (Glc) was used to rapidly prepare functional polymeric colorants in an alkaline condition in 60 min, and the resulting colorants were applied to dye silk fibers at 90 °C for 120 min. Compared with a previous report [6], the present study greatly shortened the time for the preparation of the colored silk. Another objective of this present work was to provide an approach to prepare the bioactive silk materials. In this report, the conditions for the preparation of colorants were discussed, and the characterization of colorants by ultraviolet-visible (UV–Vis) and Fourier transform infrared (FT-IR) absorption spectroscopies was performed. Furthermore, the conditions for the dyeing of silk fabric with the PGA/Glc Maillard reaction products were determined. Finally, the assessments of the color fastness, morphological structure, and antibacterial and antioxidant bioactivities of the dye silk were carried out.

## 2. Materials and Methods

### 2.1. Materials

The cosmetic grade polyglutamicacid (PGA) with a molecular weight ranging from 70 to 100 kDa (Nanjing Shineking Biotech Co. Ltd., Nanjing, China) and glucose (Glc) (Sinopharm Chemical Reagent Co. Ltd., Shanghai, China) were used. Sodium hydroxide and sulfuric acid were of analytical reagent grade. A water-soluble reactive UV absorber (UV-Sun Cel Liq.), which is based onoxalanilide and contains one vinylsulfonegroup [10,11], was kindly provided by Huntsman International LLC., Woodlands, TX, USA. The 2,2′-azino-bis (3-ethylbenzothiazoline-6-sulphonic acid) diammonium salt (ABTS) was bought from Shanghai D & B Chemicals Technology Co. Ltd., Shanghai, China. The scoured silk fabric of crepe de Chine (warp and weft count, 23.3 dtex/2; warp density, 42 threads/cm, and weft density, 60 threads/cm; weight per unit area, 52 g/m^2^) was purchased from Suzhou Jiaduoli Silk Apparel Co. Ltd., Suzhou, China.

### 2.2. Maillard Reaction of PGA and Glc

The reaction of PGA and Glc was conducted in an alkaline medium whose initial pH was adjusted using diluted sodium hydroxide. The total concentration of PGA and Glc was kept at 0.8 g/100mL. The PGA and Glc mixture was heated from 20 °C to the desired temperature at a rate of 5 °C/min, and at this temperature, the reaction continued for the desired time. Four reaction factors, including time, weight ratio of PGA and Glc, initial pH, and temperature were studied, and the detailed conditions are shown in Table 1. The optimized conditions were as follows: PGA/Glc weight ratio, 1:1; initial pH, 12; temperature, 90 °C; time, 60 min; the as-prepared reaction products whose concentration unit was labeled as g/100 mL (PGA or Glc) were used for the instrumental characterization and the dyeing of silk. Additionally, in order to improve the light stability of the PGA/Glc reaction products, in the late stage of the PGA and Glc reaction, a reactive UV absorber (0.2 g/100 mL UV-Sun Cel) was added, and the reaction continued for 30 min. Thus, novel light-stable reaction products were obtained.

### 2.3. Dyeing of Silk with the PGA/Glc Reaction Products

The PGA/Glc reaction products prepared in the optimized conditions (see Section 2.2) were employed to dye silk after their initial pH values were adjusted to between 2 and 6 by addition of dilute sulfuric acid. All the dyeing was conducted at constant temperatures in sealed and conical flasks placed in the XW-ZDR low-noise oscillated dyeing machine (Jingjiang Xinwang Dyeing and Finishing Machinery Factory, Jingjiang, China). The liquor ratio was 50:1. The influence of four factors, including initial pH, dyeing temperature, dyeing time, and PGA or Glc concentration used in the preparation of the Maillard reaction products on the color depth of silk fabric were discussed according to the experimental design shown in Table 2. At the end of dyeing, the fabrics were thoroughly rinsed in tap water and then dried in the open air.

### 2.4. Measurements

The Maillard reaction of PGA and Glc was monitored using the Shimadzu UV-1800 UV-Vis spectrophotometer (Shimadzu Co., Kyoto, Japan); the reaction stock solution (5 mL) was diluted to 10 times by distilled water, and then the spectroscopic analysis was conducted. The FT-IR spectrum of the reaction products was recorded by the Nicolet 5700FT-IR spectrometer (Thermo Fisher Scientific Inc., Waltham, MA, USA) using KBr pellets. The color depth (*K/S*), lightness (L*), redness-greenness index(a*), yellowness-blueness index (b*), and chroma (C*) of the dyed silk fabrics were measured using the HunterLabUltraScan PRO reflectance spectrophotometer (Hunter Associates Laboratory, Inc., Reston, VA, USA) using illuminant D65 and 10° standard observer. The exhaustion percentage of the Maillard reaction products on silk fiber was calculated by the difference in the initial and final absorbance of these products in solution, and their adsorption quantity was determined by the exhaustion, as well as the weight of silk fiber. The color fastness of the dyed silk to washing, rubbing, and light was tested according to ISO 105-C06, ISO 105-X12, and GB/T 8427-2008, respectively. The surface morphology of silk fabrics was observed using the Hitachi S-4800 scanning electron microscope (SEM) (Hitachi High Technologies America, Inc., Schaumburg, IL, USA).

The antibacterial activity of silk fabrics against *Staphylococcus aureus* (*S. aureus*) and *Escherichia coli* (*E. coli*) was assessed according to GB/T 20944.3-2008 (Textiles-Evaluation for Antibacterial Activity) using the shake flask method, where the standard cotton fabric was used as a reference. The antioxidant activity of silk fabrics was determined using the ABTS radical decolorization assay according to our previous method [12,13]. More details about the assessment of antibacterial and antioxidant properties are found in our previous reports [12,13]. The washing durability of the antibacterial and antioxidant properties of silk fabrics subjected to 5 and 10 washing cycles was evaluated; each washing was carried out at 40 °C for 30 min.

## 3. Results and Discussion

### 3.1. Maillard Reaction of PGA and Glc

**Figure 1:**
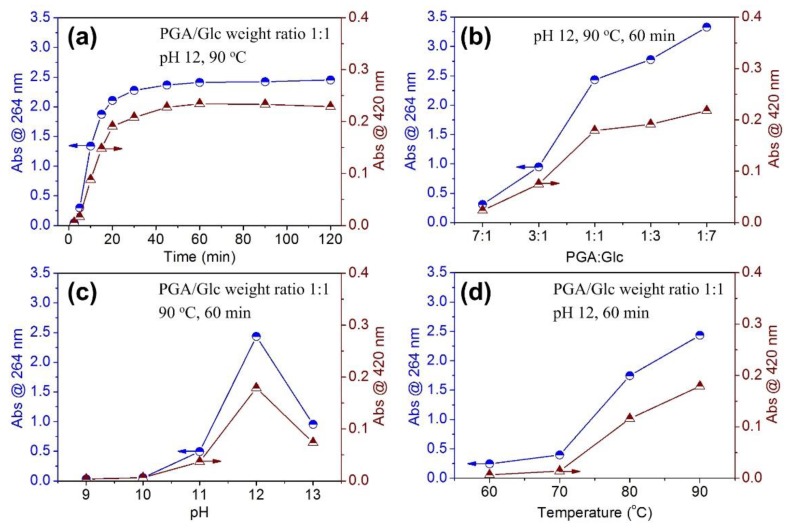
Dependence of the absorbance at 264 and 420 nm of the PGA/Glc reaction products on reaction time (**a**), weight ratio of PGA to Glc (**b**), pH (**c**), and temperature (**d**) in the presence of 0.8 g/100 mL PGA+Glc.

The PGA and Glc reaction products had the maximum absorption at 264 nm, and displayed some absorption in the visible region. These absorptions were related to the formation of reaction products. In this work, the absorbance at 264 and 420 nm was monitored. Figure 1 reveals the effects of four reaction factors on the absorbance at 264 and 420 nm. The reaction of PGA and Glc approached equilibrium at 60 min as longer time did not cause increasing absorbance (Figure 1a). The absorbance at both 264 and 420 nm increased remarkably with the Glc content (Figure 1b), indicating the significant role of reducing sugar on the generation of the browning colored products [14]. As expected, the Maillard browning increased with pH [1,15]. At pH 12, the absorbance reached a maximum (Figure 1c). However, increasing the pH to 13 led to a remarkably reduced absorbance, which may be attributed to the side reaction occurring between PGA and Glc in the presence of excess alkali, reducing the quantity of Maillard reaction products. Figure 1d shows that the reaction of PGA and Glc proceeded more completely at 80 and 90 °C than at 60 and 70 °C, revealing that the reaction is rapid at high temperature. Based on the above results, the colored products were prepared with PGA and Glc equal in weight at 90 °C and pH 12 for 60 min.

### 3.2. Characterization of the Maillard Reaction Products

**Figure 2:**
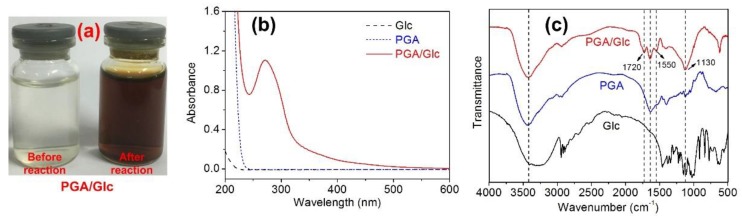
Color (**a**),UV-Vis adsorption spectra (**b**), and FT-IR spectra (**c**) of the PGA/Glc reaction products, PGA and Glc.

Figure 2a shows the color change of a PGA and Glc mixture before and after reaction, from which the dark brown color products were observed. Figure 2b shows the characteristic absorption band of the reaction solution at around 264 nm, indicating the formation of melanoidin colorants from the Maillard reaction [15,16]. Figure 2c shows that the FT-IR spectrum of the colored products was completely different from those of PGA and Glc. Compared with the spectra of pure PGA and Glc, the new bands corresponding to the C=O (1720 cm^−1^) and C=N (1550 cm^−1^) were observed in the FT-IR spectrum of the reaction products, indicating that the Schiff base was yielded by reaction of the reducing end of Glc and the amino groups of PGA [17,18]. The prominent band at 1130 cm^−1^ of the reaction products was attributed to the C–O, which is present in the structure of melanoidins [19]. The above observations demonstrate the formation of the colored melanoidin products from the Maillard reaction of PGA and Glc.

### 3.3. Dyeing of Silk with the PGA/Glc Reaction Products

**Figure 3:**
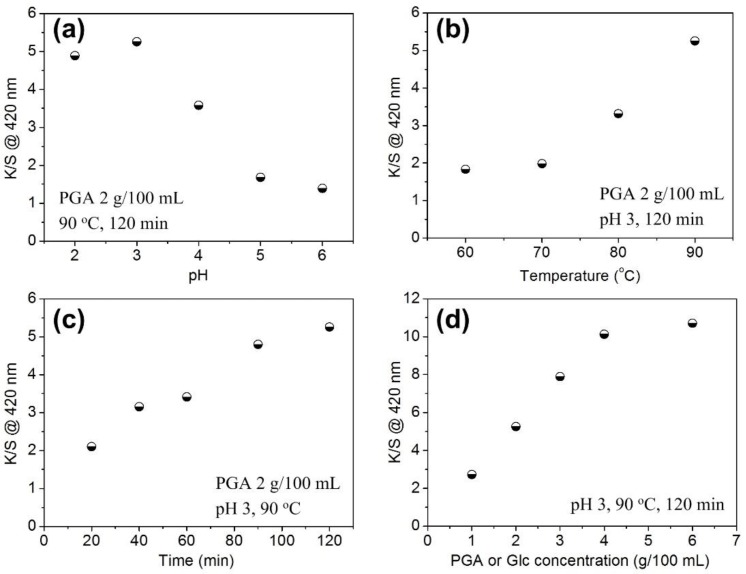
Dependence of the color depth of silk fabrics dyed with the PGA/Glc reaction products on pH (**a**), temperature (**b**), time (**c**), and PGA or Glc concentration (**d**) in the case of a PGA/Glc weight ratio of 1:1.

Figure 3 shows the color depth of silk fabric dyed with the PGA/Glc reaction products under different conditions. All the four dyeing parameters had remarkable impact on the color depth of silk fabric. The color depth reached a maximum at pH 3, and then decreased as pH increased (Figure 3a). This finding revealed that the electrostatic interactions between the dissociated carboxyl groups in the PGA/Glc reaction products and the protonated amino groups in silk fiber contribute to the dyeing effects. Too low a pH inhibits the ionization of carboxyl groups in the PGA/Glc reaction products, and high pH lowers the protonation of amino groups in silk fiber. Both of them negatively affect the adsorption of the PGA/Glc reaction products on silk fiber, and thereby decrease the color depth of silk fabric.

Figure 3b shows that the application of the PGA/Glc reaction products had an interesting temperature effect. The color depth of silk fabric was very low below 70 °C, but it increased markedly at higher temperatures. This phenomenon was initially thought to be associated with the high molecular weight of the PGA/Glc reaction products. The adsorption of macromolecules on fibers usually exhibits an endothermic effect [20,21], and their adsorption extent increases with an increase in temperature. Figure 3c shows that the color depth of silk fabric increased with the extension of time, as expected, and a high color depth required a long dyeing time. Figure 3d shows a gradual increase in the color depth of silk fabric with increasing initial concentration of PGA or Glc. At a PGA or Glc concentration higher than 4 g/100 mL, the color depth had a very limited increase, indicating that the deposition of the PGA/Glc reaction products on silk fiber approaches saturation.

**Figure 4:**
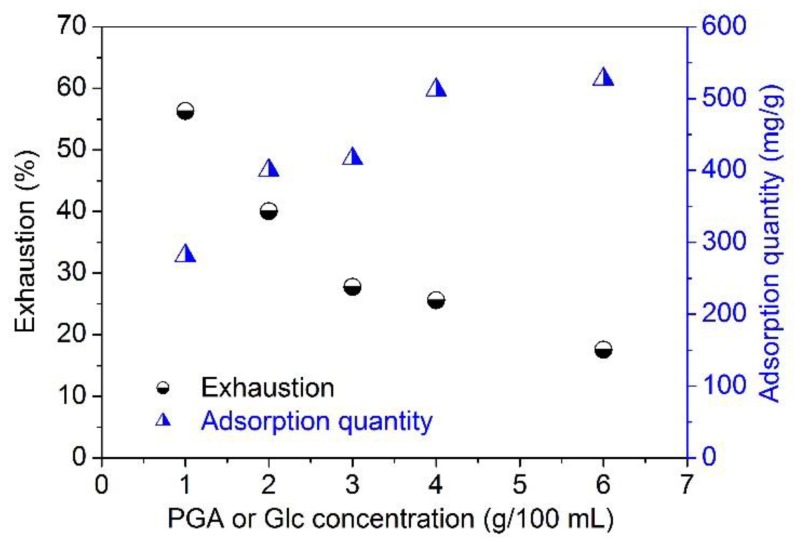
Exhaustion and adsorption quantity of the PGA/Glc reaction products at various concentrations on silk fabrics.

Figure 4 shows the exhaustion of the PGA/Glc reaction products. As the concentration of the products increased, their exhaustion decreased gradually. On the other hand, the quantity of their adsorption by silk increased progressively with an increase in the concentration of the products, and approached saturation at a concentration of 4 g/100 mL, which is in good agreement with the results of Figure 3d. The products did not display high exhaustion on silk fiber. This might be associated with the high quantity of the products. At a low concentration (e.g., 1–2 g/100 mL), the products showed relatively high exhaustion (Figure 4), while it yielded a pale coloration on silk (Figure 3d). However, a low concentration of products was able to impart good antibacterial and antioxidant functions to silk fiber (see Section 3.6). Therefore, if the PGA/Glc reaction products are put into use, their concentrations can be reasonably determined according to the requirements for the color depth and functions of silk fiber. Additionally, the approaches to increase the exhaustion of the PGA/Glc reaction products deserve to be studied.

### 3.4. Color Characteristics and Color Fastness of the Dyed Silk

**Figure 5:**
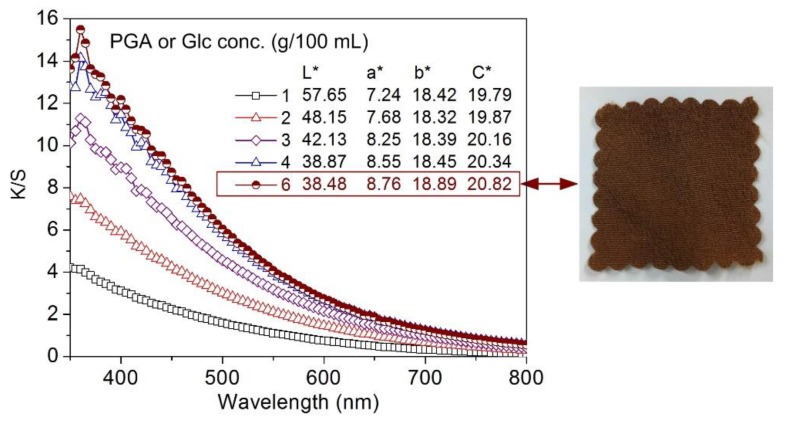
Spectral data of silk fabrics dyed with the PGA/Glc reaction products at various concentrations (The inserted photo represents the dyed silk sample).

Figure 5 shows the color characteristics of silk fabrics dyed with the PGA/Glc reaction products at various concentrations. All the dyed samples exhibited low a*, b*, and C*values, revealing their dull color nature. Positive b* values higher than positive a* values demonstrated that the colors of the dyed samples were yellowish-brown. An example is given in the inserted photo of Figure 5, which represents the dark brown color of the sample dyed with the 6 g/100 mL PGA/Glc reaction products. The L* values decreased with an increase in the concentration of the PGA/Glc reaction products, which was in agreement with the increasing *K/S* values. Moreover, no sharp absorption band was found for the dyed samples in the visible light region. This optical absorption characteristic is greatly different from those of synthetic dyes, which have sharp absorption bands, implying that there are no strongly conjugated π-electron systems in the colored melanoidin products derived from the Maillard reaction of PGA and Glc.

Table 3 lists the color fastness of silk fabrics dyed with the PGA/Glc reaction products (6 g/100 mL). The washing and rubbing fastness had high levels, implying the strong interactions between melanoidin colorants and silk fiber. According to GB 18401–2010: National General Safety Technical Code for Textile Products (Chinese National Standards for Textiles) [22], the baby/children products have the following requirements: washing fastness ≥3–4 and rubbing fastness ≥4, while the washing and rubbing fastness of the products direct and indirect contact with skin should be greater than or equal to 3. The fastness ratings of the dyed silk fabric were above the Chinese National Standard for the acceptable fastness to washing and rubbing. However, the light fastness was poor, revealing that the color products are unstable to light. In order to improve the light fastness, in the late stage of the PGA and Glc reaction, a reactive UV absorber (UV-Sun Cel) was added, and it was able to react with the hydroxyl groups of the melanoidin colorants. Thus, the novel melanoidin colorants were obtained. The modification of melanoidin colorants with the reactive UV absorber had almost no impact on the color hue and color depth of silk fabric, but it increased the light fastness from 2 to 3–4, as shown in Table 3.

### 3.5. Morphological Structure of the Dyed Silk

**Figure 6:**
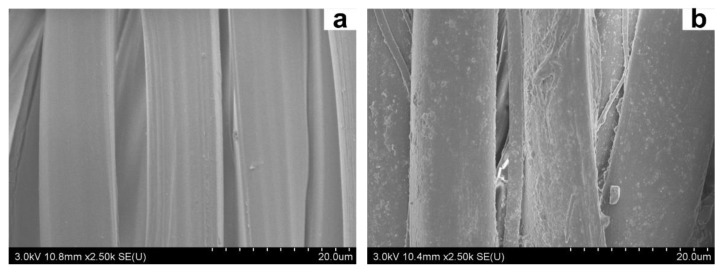
SEM micrographs of the undyed (**a**) and dyed (**b**) silk fabrics.

The surface morphology of silk fabrics dyed without and with the PGA/Glc reaction products (6 g/100 mL) was observed by SEM. As shown in Figure 6, the undyed silk fiber displayed a smooth and clean surface, whereas on the surface of the dyed sample, the deposition of polymeric substances was found. These surface substances should be the melanoidin colorants with high molecular weights. According to the SEM analysis, it was concluded that the surface coloration occurs for the dyeing of silk fiber with the PGA/Glc reaction products. Also, it was initially thought that the strong adhesive forces of the PGA/Glc reaction products on the silk surface contribute to the excellent washing and rubbing color fastness of the dyed silk.

### 3.6. Antibacterial and Antioxidant Activity of the Dyed Silk

**Figure 7:**
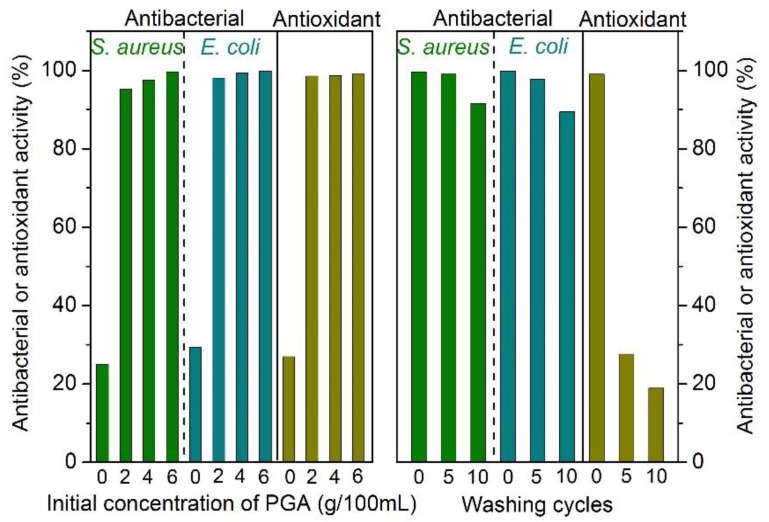
Antibacterial and antioxidant activity of the dyed silk fabrics and their washing durability for the dyed silk with the 6 g/100 mL PGA/Glc reaction products.

**Figure 8:**
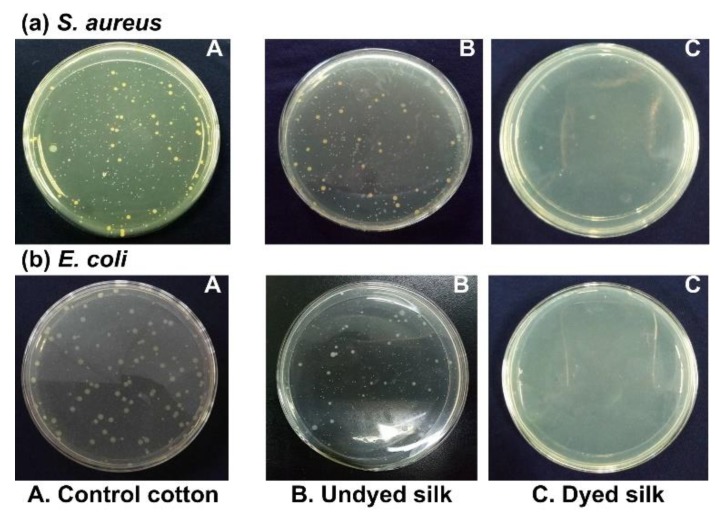
Visual bacterial cultures for the control cotton (**A**), the undyed silk (**B**), and the dyed silk with the 6 g/100 mL PGA/Glc reaction products (**C**).

From Figure 7, it is clear that all the dyed silk fabrics exhibited excellent antibacterial and antioxidant activity, and moreover, the antibacterial activity had a slight increment with increasing initial concentration of the PGA/Glc reaction products. Figure 8 also shows the significant differences of the visual bacterial cultures between the undyed and dyed silk samples. For the dyed sample, almost no bacterial colonies were observed, revealing its excellent antibacterial behavior. The washing durability of the antibacterial activity was very good; even after 10 washing cycles, the antibacterial activity still reached 90% or so. Unexpectedly, the antioxidant activity had a very poor washing resistance.

The antibacterial activity of the dyed silk fabrics may have stemmed from PGA and melanoidins derived from the PGA and Glc reaction. PGA itself can behave as an antibacterial agent [23], and melanoidins also possess good antibacterial ability [2]. The high antibacterial activity of the fabric subjected to repeated washing might have been associated with the antibacterial ability of PGA itself. It is worth pointing out that melanoidin compounds can contribute to the antioxidant activity of the dyed silk fabrics. Although the specific components responsible for the antioxidant ability of melanoidins are not yet known, the radical-scavenging activity of melanoidins has been proved by a large of amount of literature [2]. The low antioxidant activity of the fabric subjected to repeated washing was likely due to the fact that the melanoidin components possessing antioxidant ability are washed off in the washing process.

## 4. Conclusions

The Maillard reaction of PGA and Glc in an alkaline condition was successfully utilized to rapidly prepare the colored compounds. The UV-Vis spectroscopic analysis revealed that the quantity of the colored products generated was greatly dependent on the weight ratio of PGA to Glc, pH, and temperature. The optimized conditions for the generation of colored products were as follows: PGA/Glc weight ratio 1:1, pH 12, and 90 °C. The resulting products containing melanoidins were confirmed by the FT-IR analysis. After being dyed with the PGA/Glc reaction products, the silk fiber was covered by polymeric layers, and exhibited very good fastness to washing and rubbing. A shortcoming of the dyed silk was poor light fastness, but it could be improved by means of incorporating the UV absorbing moiety into the PGA/Glc reaction products. The dyed silk possessed good and durable antibacterial activity, and had good initial antioxidant activity, but the antioxidant activity had a poor resistance to washing. The present research provides a novel approach to obtain the colored and functional silk materials by rapidly preparing the Maillard reaction products of PGA and Glc, and then applying them using a conventional dyeing technique.

## Figures and Tables

**Table 1:** Reaction conditions of PGA and Glc.

Variable	Levels	Other p-arameters
Time	2.5 to 120 min	PGA/Glc weight ratio 1:1, pH 12, 90 °C
PGA/Glc weight ratio	7:1 to 1:7	pH 12, 90 °C, 60 min
pH	9 to 13	PGA/Glc weight ratio 1:1, 90 °C, 60 min
Temperature	60 to90°C	PGA/Glc weight ratio 1:1, pH 12, 60 min

**Table 2:** Dyeing conditions of silk with the PGA/Glc reaction products.

Variable	Levels	Other parameters
pH	2–6	PGA 2 g/100mL, 90 °C, 120 min
Temperature	60–90°C	PGA 2 g/100mL, pH 3, 120 min
Time	20–120 min	PGA 2 g/100 mL, pH 3, 90 °C
PGA concentration	1–6 g/100 mL	pH 3, 90 °C, 120 min

**Table 3:** Color fastness of silk fabrics dyed with the PGA/Glc reaction products.

Reactionproducts	Washing (rating)			Rubbing (rating)		Light (rating)
	Color change	Stain		Dry	Wet	
		Silk	Cotton			
No UV-Sun Cel	5	4–5	4–5	5	4–5	2
UV-Sun Cel	5	4–5	4–5	5	4–5	3–4
